# Synthesis, Characterization and *In Vitro* Anticancer Activity of C-5 Curcumin Analogues with Potential to Inhibit TNF-*α*-Induced NF-*κ*B Activation

**DOI:** 10.1155/2014/524161

**Published:** 2014-07-24

**Authors:** Amit Anthwal, Bandana K. Thakur, M. S. M. Rawat, D. S. Rawat, Amit K. Tyagi, Bharat B. Aggarwal

**Affiliations:** ^1^Department of Chemistry, H.N.B. Garhwal University, Srinagar Garhwal, Uttarakhand 246174, India; ^2^Department of Chemistry, University of Delhi, Delhi 110007, India; ^3^Cytokine Research Laboratory and Pharmaceutical Development Center, Department of Experimental Therapeutics, The University of Texas M. D. Anderson Cancer Center, Houston, Texas 77030, USA

## Abstract

In a search of new compounds active against cancer, synthesis of a series of C-5 curcumin analogues was carried out. The new compounds demonstrated good cytotoxicity against chronic myeloid leukemia (KBM5) and colon cancer (HCT116) cell lines. Further, these compounds were found to have better potential to inhibit TNF-*α*-induced NF-*κ*B activation in comparison to curcumin, which show their potential to act as anti-inflammatory agents. Some compounds were found to show higher cytotoxicity against cancer cell lines in comparison to curcumin used as standard.

## 1. Introduction

**Figure 1:**
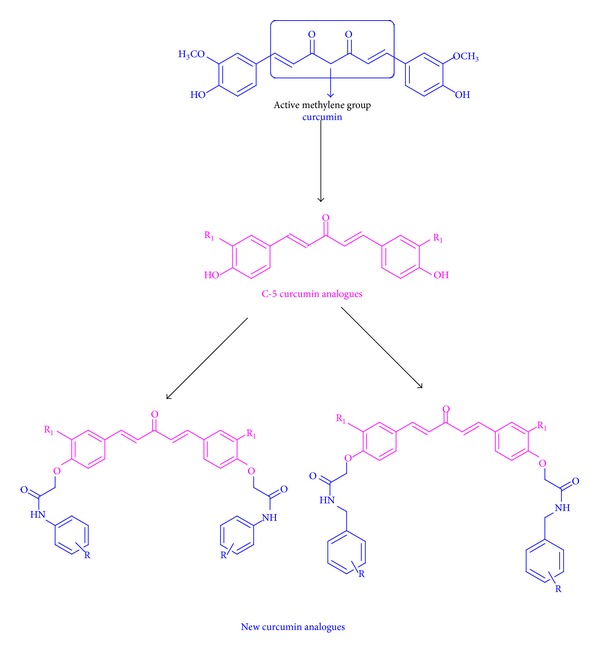
Modification of curcumin to get new C-5 curcumin analogues.

In the last few decades, importance has been given to biologically active natural products as these compounds generally do not have any side effects. The proof of this is the fact that more than 62% of the anticancer drugs approved from 1983 to 1994 are either natural products or natural product analogues [1]. Curcumin and related compounds, termed as curcuminoid, are among the compounds of great interest due to their wide range of biological activities [2–12]. Curcumin ability to inhibit the growth of various types of cancer cells at various stages of cancer progression is due to its potential to act on multiple targets [13–17]. The *β*-diketone is the structural feature responsible for rapid metabolism of curcumin by aldo-keto reductase in liver [18]. Tremendous research work has been done to improve the bioavailability and absorption of curcumin [19]. Further,* in vivo* and* in vitro* studies showed that curcumin undergo rapid metabolism by oxidation, reduction, glucuronidation, and sulfation [20, 21], which occur at 4-OH [22]. In a rational approach to design new curcumin analogues the facts to be taken under consideration are modification of *β*-diketone moiety and blocking 4-OH of curcumin analogues. In efforts to improve the activity and stability of curcumin analogues, C-5 curcumins have been designed and synthesized by various research groups [23, 24]. It has been reported that C-5 curcumin analogues show better activity and stability in* in vitro* and* in vivo* studies [25]. Yamakoshi et al. have done SAR studies on C-5 curcumin analogues. Important outcomes of their studies were that symmetry is important in case of tetrasubstituted C-5 curcumin analogues and 4-position is a possible site for attaching probe to enhance activity [26]. In search of new molecules with good cytotoxicity against cancer cells we planned to synthesize new C-5 curcumin analogues and selected amido-ether linker for blocking 4-OH (Figure 1).

As a part of our research work towards development of biologically important hybrid molecules [27], we designed new curcumin analogues. In present work, we report synthesis, theoretical prediction of physicochemical properties, cytotoxicity, and inhibition of TNF-*α*-induced NF-*κ*B activation of C-5 curcumin analogues (**3a**–**3p**) in human cancer cell lines. New hybrid molecules demonstrated varying level of cytotoxicity against KBM5 and HCT116 cancer cell lines with some compounds being more active than curcumin against both cell lines. Also compounds were found to inhibit TNF-*α*-induced NF-*κ*B activation.

## 2. Results and Discussion

### 2.1. Chemistry

**Scheme 1:**
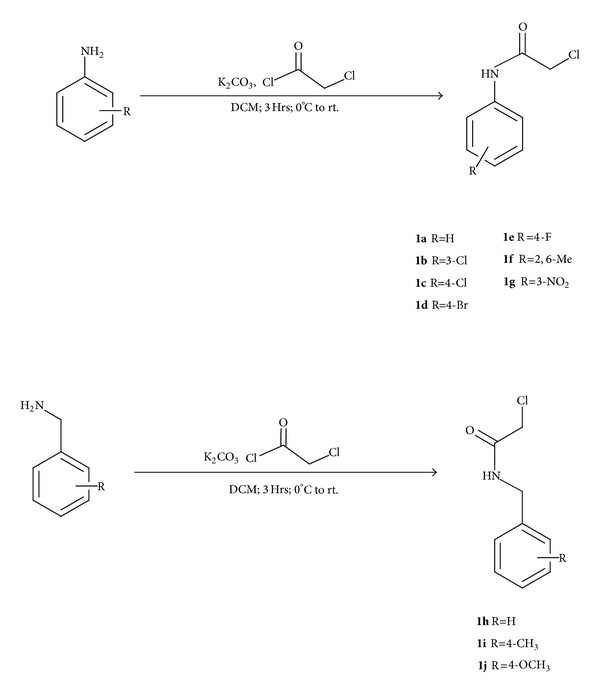


**Scheme 2:**
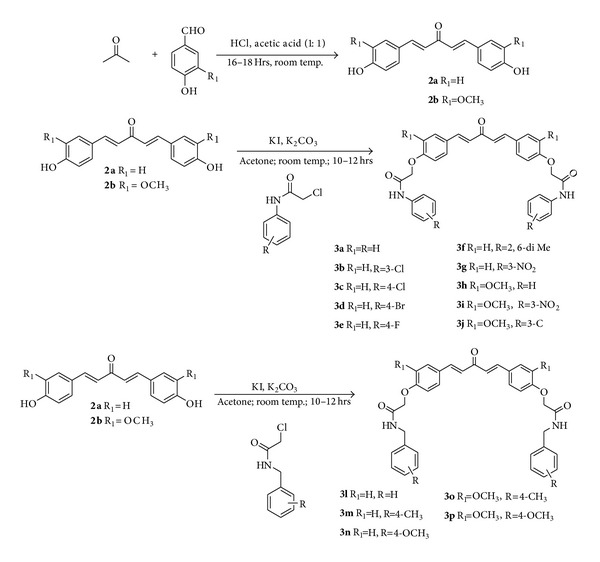


The C-5 curcumin analogues were synthesized by multistep synthesis process. The amido-ether linkers (**1a**–**1j**) were synthesized by reaction of respective aromatic amines/benzyl amines with chloroacetyl chloride in the presence of K_2_CO_3_ as base and dichloromethane as solvent (Scheme 1). C-5 curcumin analogues (**2a**,** 2b**) were synthesized by reaction of 4-hydroxybenzaldehyde/vanillin with acetone in the presence of 1 : 1 acetic acid/HCl solvent as well as catalyst. The C-5 curcumin analogues (**2a**,** 2b**) were further reacted with amido-ether linkers (**1a**–**1j**) in the presence of K_2_CO_3_ as base and acetone as solvent to obtain the desired hybrid molecules (**3a**–**3p**) in good yields (Scheme 2).

### 2.2. Biology

**Figure 2:**
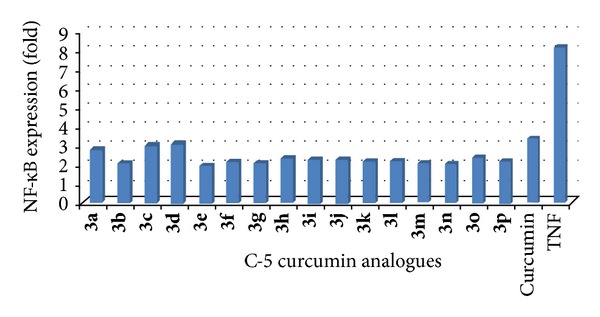
Downregulation of TNF-*α*-induced NF-*κ*B activation in KBM5 cells. KBM-5 cells were incubated with 5 *μ*M dose of tested compounds for 8 h and then treated with 0.1 nM TNF-*α* for 30 min. Nuclear extracts were prepared and assayed for NF-*κ*B activation using EMSA. The fold downmodulation of NF-*κ*B as compared to TNF-*α* is shown.

All C-5 curcumin analogues were tested for their cytotoxicity on chronic myeloid leukemia (KBM5) and colon cancer (HCT116) cell lines. However, the cytotoxicity pattern of all analogues was similar for both cell lines (Table 1). In C-5 curcumin nucleus variations were done by substituting  –H by –OMe whereas in amido-ether linker substituted aromatic amide and benzylamide derivatives were used. In the case of curcumin analogues with aromatic amides (**3a**–**3k**), halogen substituents did not give fruitful results (**3c**,** 3d**,** 3e**) except for compounds** 3b** and** 3k** with chloro substituent at 3 and 4 positions, respectively, which demonstrated cytotoxicity better than curcumin in both cancer cell lines. The compounds without any substitution (**3a**,** 3h**) were found to be more active than the curcumin but the compound with −OMe group on C-5 curcumin ring (**3h**) was found most active. Substitution by nitro group in aromatic ring (**3g**,** 3i**) resulted in compounds with poor activity. Substitution by methyl groups at the 2,6-positions gave promising results in the case of molecule with –OMe substituent on C-5 curcumin ring (**3j**) but molecule without any substituent in C-5 curcumin ring demonstrated poor activity (**3f**). In case of curcumin analogues with benzylamide derivatives the hybrid molecule with  –OMe substitution in aromatic ring as well as C-5 curcumin nucleus demonstrated potent activity (**3p**) whereas other compounds without any substituent (**3l**) or with –Me (**3m**,** 3o**) and –OMe (**3n**) substituents on aromatic ring exhibited poor cytotoxicity. The newly synthesized compounds were also screened for their potential to inhibit TNF-*α*-induced NF-*κ*B activation (Figure 2). All molecules demonstrated higher potential to inhibit TNF-*α*-induced NF-*κ*B activation in comparison to curcumin from which it could be concluded that these compounds can act as very good anti-inflammatory agents.

### 2.3. Theoretical Predictions of Physicochemical Properties [28–31]

Molinspiration and Osiris software were used for theoretical prediction of physicochemical properties of the new hybrid molecules. The methodology developed by molinspiration is used to calculate *m*_*i*_log⁡*P* (octanol/water partition coefficient). Total polar surface area (TPSA) has been reported to be a very good descriptor of various characteristics of compound such as absorption, including intestinal absorption, bioavailability, Caco-2 permeability, and blood brain barrier penetration. Theoretical molecular properties, predicted by molinspiration software, for new C-5 curcumin analogues (**3a**–**3p**) are tabulated in Table 2. The values of lipophilicity (log *P*) and total polar surface area (TPSA) are two important parameters for the prediction of oral bioavailability of drug molecules [32, 33]. It has been reported that molecules with TPSA values of 140 Å^2^ or more are likely to exhibit poor intestinal absorption [33].

Theoretical molecular properties, predicted by Osiris software, for new C-5 curcumin analogues (**3a**–**3p**) are tabulated in Table 3 which include toxicity risks (mutagenicity, tumorigenicity, irritation, and reproduction) and physicochemical properties (*m*_*i*_ log *P*, solubility, drug likeness, and drug score) of compounds (**3a**–**3p**). Toxicity risk alerts give probability of harmful risks under specified category. From data in Table 3 it is clear that most of the molecules are supposed to be nonmutagenic, nonirritating with no reproductive effects. The drug score is sum of various parameters such as drug likeness, *m*_*i*_ Log *P*, log *S*, molecular weight, and toxicity risks in the form of single valued figure that may be used to judge the compounds overall capability to qualify requirements for a drug. It was found that most of the compounds (**3a**–**3p**) have properties comparable to that of the standard compound curcumin.

## 3. Experimental Section

### 3.1. General

All chemicals used in synthesis were purchased from Sigma-Aldrich and Himedia. Thin layer chromatography (Merck TLC silica gel 60 F_254_) was used to monitor the progress of reactions. The compounds were purified when needed by silica gel column (60–120 meshes). Melting points were determined on EZ-Melt automated melting point apparatus, Stanford Research systems, and are uncorrected. IR (chloroform/film) spectra were recorded using Perkin-Elmer FT-IR spectrophotometer and values are expressed as *υ*_max⁡_ cm^−1^. Mass spectra were recorded in waters micromass LCT Mass Spectrometer. The ^1^H NMR and ^13^C NMR spectra were recorded on Jeol ECX spectrospin at 400 MHz and 100 MHz, respectively, in deuterated solvents with TMS as an internal standard. Chemical shift values are recorded on *δ* ppm and the coupling constants *J* are in Hz.

### 3.2. General Procedure for Synthesis of N-Phenyl and N-Benzyl Acetamides (**1a**–**1j**)

To a stirred solution of respective aromatic amine derivatives/benzyl amine derivatives (10 mmol) in dichloromethane, 30 mmol of K_2_CO_3_ was added. The reaction mixture was cooled to 0°C and chloroacetyl chloride (11 mmol) was added slowly drop wise. After addition of chloroacetyl chloride reaction mixture was allowed to stir at room temperature for 3 hours. After completion of reaction solvent was evaporated with rota evaporator and residue obtained was filtered and washed thoroughly with water. The product obtained (**1a**–**1j**) was pure enough to be used as such in subsequent steps.

### 3.3. General Procedure for Synthesis of C-5 Curcumin Analogues (**2a**–**2b**)

To a stirred solution of acetone (30 mmol) in 1 : 1 acetic acid/HCl p-hydroxybenzaldehyde/vanillin (63 mmol) was added, respectively. The reaction mixture was allowed to stir for 16–18 hours at room temperature. After completion of reaction, the product was precipitated by addition of water to reaction mixture. The precipitate obtained was filtered, washed with water, and recrystallized from ethanol to get pure compound (**2a**,** 2b**) in good yield.

### 3.4. General Procedure for Synthesis of New C-5 Curcumin Analogues (**3a**–**3p**)

To a stirred solution of C-5 curcumin analogue (**2a**/**2b**) (0.84 mmol) in acetone, 0.25 mmol of KI and 2.52 mmol of K_2_CO_3_ were added. Further, 1.7 mmol of respective amide (**1a**–**1j**) was added to reaction mixture and it was allowed to stir at room temperature for 10–12 hours. After completion of reaction, monitored by TLC, the solvent was evaporated and residue obtained was filtered and washed with water. The crude product obtained was purified by column chromatography using ethyl acetate/hexane as eluent to get desired compounds in good yield (**3a**–**3p**).

#### 3.4.1. 2, 2′-(((1E,4E)-3-Oxopenta-1,4-diene-1,5-diyl)bis(4,1-phenylene))bis(oxy)bis(N-zhenylacetamide) **3a**

Yield 80% (yellow solid); m.p. 193–195°C; IR (KBr film) *υ*_max⁡_ cm^−1^: 3377, 3058, 2914, 1679, 1649, 1599, 1534, 1507, 1442, 1328, 1248, 1173, 1098, 1058, 984, 837, and 755; ^1^H NMR (DMSO-*d*_6_, 400 MHz): *δ* 4.77 (s, 4H), 7.06 (t, 3H, *J* = 3.7 Hz), 7.08 (d, 3H, *J* = 2.2 Hz), 7.19 (d, 1H, *J* = 15.4 Hz), 7.29 (d, 2H, *J* = 7.3 Hz), 7.32 (d, 3H, *J* = 7.3 Hz), 7.42 (d, 1H, *J* = 6.6 Hz), 7.54 (d, 1H, *J* = 8.8 Hz), 7.62 (d, 3H, *J* = 7.3 Hz), 7.71 (d, 2H, *J* = 15.4 Hz), 7.75 (d, 3H, *J* = 8.8 Hz), and 10.11 (brs, 2H); TOF-MS* m/z*: 533.1998 (M + 1), calculated for C_33_H_28_N_2_O_5_: 532.1878.

#### 3.4.2. 2, 2′-((((1E,4E)-3-Oxopenta-1,4-diene-1,5-diyl)bis(4,1-phenylene))bis(oxy))bis(N-(3-chlorophenyl)acetamide) **3b**

Yield 87% (yellow solid); m.p. 206–208°C; IR (KBr film) *υ*_max⁡_ cm^−1^: 3400, 2914, 1678, 1651, 1593, 1508, 1423, 1244, 1173, 1071, 996, 830, and 772; ^1^H NMR (DMSO-*d*_6_, 400 MHz): *δ* 4.78 (s, 4H), 7.06 (d, 4H, *J* = 8.1 Hz), 7.12 (d, 2H, *J* = 8.1 Hz), 7.19 (d, 2H, *J* = 15.4 Hz), 7.33 (t, 2H, *J* = 8.1 Hz), 7.37–7.46 (m, 1H), 7.53 (t, 2H, *J* = 8.1 Hz), 7.69 (d, 1H, *J* = 16.8 Hz), 7.74 (d, 4H, *J* = 8.8 Hz), and 7.82 (s, 2H); TOF-MS* m/z*: 601.1219 (M + 1), calculated for C_33_H_26_Cl_2_N_2_O_5_: 600.1345.

#### 3.4.3. 2, 2′-((((1E,4E)-3-Oxopenta-1,4-diene-1,5-diyl)bis(4,1-phenylene))bis(oxy))bis(N-(4-chlorophenyl)acetamide) **3c**

Yield 85% (light yellow solid); m.p. 219–221°C; IR (KBr film) *υ*_max⁡_ cm^−1^: 3330, 2929, 1672, 1643, 1599, 1508, 1400, 1249, 1170, 1093, 1009, 974, 833, and 702; ^1^H NMR (*δ*_6_-DMSO, 400 MHz): *δ* 4.77 (s, 4H), 7.07 (d, 4H, *J* = 8.8 Hz), 7.20 (d, 2H, *J* = 15.4 Hz), 7.35 (d, 2H, *J* = 5.1 Hz), 7.38 (d, 2H, *J* = 5.1 Hz), 7.44–7.51 (m, 1H), 7.65 (d, 2H, *J* = 5.1 Hz), 7.67 (d, 2H, *J* = 2.9 Hz), 7.68 (d, 1H, *J* = 8.8 Hz), and 7.75 (d, 4H, *J* = 8.8 Hz); TOF-MS* m/z*: 601.1219 (M+1), calculated for C_33_H_26_Cl_2_N_2_O_5_: 600.1345.

#### 3.4.4. 2, 2′-((((1E,4E)-3-Oxopenta-1,4-diene-1,5-diyl)bis(4,1-phenylene))bis(oxy))bis(N-(4-bromophenyl)acetamide) **3d**

Yield 87% (yellow solid); m.p. 210–212°C; IR (KBr film) *υ*_max⁡_ cm^−1^: 3385, 2927, 1689, 1644, 1602, 15.08, 1397, 1244, 1172, 1065, 1008, 831, and 703; ^1^H NMR (DMSO-*d*_6_,400 MHz): *δ* 4.77 (s, 4H), 7.06 (d, 4H, *J* = 8.8 Hz), 7.19 (d, 2H, *J* = 16.1 Hz), 7.48 (d, 4H, *J* = 8.8 Hz), 7.60 (d, 4H, *J* = 8.8 Hz), 7.71 (d, 2H, *J* = 16.5 Hz), and 7.74 (d, 4H, *J* = 8.8 Hz); TOF-MS* m/z*: 689.0208 (M + 1), calculated for C_33_H_26_Br_2_N_2_O_5_: 688.0355.

#### 3.4.5. 2, 2′-((((1E,4E)-3-Oxopenta-1,4-diene-1,5-diyl)bis(4,1-phenylene))bis(oxy))bis(N-(4-fluorophenyl)acetamide) **3e**

Yield 87% (yellow solid); m.p. 263-264°C; IR (KBr film) *υ*_max⁡_ cm^−1^: 3385, 3043, 2916, 1678, 1647, 1602, 1585, 1536, 1508, 1411, 1340, 1247, 1173, 1098, 1059, 984, 834, and 733; ^1^H NMR (DMSO-*d*_6_, 400 MHz) *δ*: 4.77 (s, 4H), 7.08 (d, 4H, *J* = 8.8 Hz), 7.15 (d, 1H, *J* = 16.1 Hz), 7.16 (d, 2H, *J* = 6.6 Hz), 7.18 (d, 1H, *J* = 5.9 Hz), 7.22 (d, 2H, *J* = 16.1 Hz), 7.65 (dd, 2H, *J*_1_ = 2.2 Hz, *J*_2_ = 2.9 Hz), 7.66 (d, 1H, *J* = 2.2 Hz), 7.67 (d, 1H, *J* = 6.2 Hz), 7.72 (d, 1H, *J* = 16.1 Hz), and 7.76 (d, 5H, *J* = 8.8 Hz); TOF-MS* m/z*: 569.1810 (M + 1), calculated for C_33_H_26_F_2_N_2_O_5_: 568.1648.

#### 3.4.6. 2, 2′-((((1E,4E)-3-Oxopenta-1,4-diene-1,5-diyl)bis(4,1-phenylene))bis(oxy))bis(N-(2,6-dimethylphenyl)acetamide) **3f**

Yield 81% (yellow solid); m.p. 225–227°C; IR (KBr film) *υ*_max⁡_ cm^−1^: 3404, 2924, 1674, 1622, 1601, 1509, 1422, 1226, 1172, 1098, 980, 826, 764, and 703; ^1^H NMR (DMSO-*d*_6_, 400 MHz): *δ* 2.11 (s, 12H), 4.81 (s, 4H), 7.07 (d, 6H, *J* = 6.6 Hz), 7.11 (d, 4H, *J* = 8.1 Hz), 7.23 (d, 2H, *J* = 16.1 Hz), 7.74 (d, 2H, *J* = 16.1 Hz), and 7.78 (d, 4H, *J* = 8.8 Hz); TOF-MS* m/z*: 589.2624 (M + 1), calculated for C_37_H_36_N_2_O_5_: 588.2354.

#### 3.4.7. 2, 2′-((((1E,4E)-3-Oxopenta-1,4-diene-1,5-diyl)bis(4,1-phenylene))bis(oxy))bis(N-(3-nitrophenyl)acetamide) **3g**

Yield 87% (yellow solid); m.p. 185–187°C; IR (KBr film) *υ*_max⁡_ cm^−1^: 3380, 3122, 2913, 1703, 1648, 1600, 1527, 1508, 1425, 1350, 1242, 1173, 1069, 974, and 830; ^1^H NMR (DMSO-*d*_6_, 400 MHz): *δ* 4.82 (s, 4H), 7.08 (d, 4H, *J* = 8.8 Hz), 7.19 (d, 2H, *J* = 15.4 Hz), 7.60 (t, 2H, *J* = 8.1 Hz), 7.72 (d, 2H, *J* = 16.1 Hz), 7.75 (d, 4H, *J* = 8.8 Hz), 7.91 (dd, 2H, *J*_1_ = 2.2 Hz, *J*_2_ = 6.6 Hz), 7.98 (d, 2H, *J* = 8.1 Hz), and 8.66 (t, 2H, *J* = 2.2 Hz); TOF-MS* m/z*: 623.1700 (M + 1), calculated for C_33_H_26_N_4_O_9_: 622.1546.

#### 3.4.8. 2, 2′-((((1E,4E)-3-Oxopenta-1,4-diene-1,5-diyl)bis(2-methoxy-4,1-phenylene))bis(oxy))bis(N-phenylacetamide) **3h**

Yield 87% (yellow solid); m.p. 208–210°C; IR (KBr film) *υ*_max⁡_ cm^−1^: 3371, 2937, 1693, 1644, 1617, 1593, 1509, 1485, 1422, 1309, 1261, 1189, 1093, 1034, 978, 826, and 720; ^1^H NMR (CDCl_3_, 400 MHz): *δ* 4.01 (s, 6H), 4.69 (s, 4H), 6.97 (d, 2H, *J* = 5.9 Hz), 7.00 (d, 2H, *J* = 1.5 Hz), 7.15 (d, 1H, *J* = 7.3 Hz), 7.17 (s, 1H), 7.19 (d, 2H, *J* = 1.5 Hz), 7.24 (dd, 2H, *J*_1_ = 1.5, *J*_2_ = 6.6 Hz), 7.35 (d, 3H,* J* = 8.1 Hz), 7.38 (s, 1H), 7.59 (d, 4H, *J* = 7.3 Hz), 7.69 (d, 2H, *J* = 16.1 Hz), and 8.75 (brs, 2H); TOF-MS* m/z*: 593.2210 (M + 1), calculated for C_35_H_32_N_2_O_7_: 592.2340.

#### 3.4.9. 2, 2′-((((1E,4E)-3-Oxopenta-1,4-diene-1,5-diyl)bis(2-methoxy-4,1-phenylene))bis(oxy))bis(N-(3-nitrophenyl)acetamide) **3i**

Yield 87% (yellow solid); m.p. 173–175°C; IR (KBr film) *υ*_max⁡_ cm^−1^: 3381, 3102, 2930, 1693, 1657, 1597, 1531, 1425, 1350, 1256, 1140, 1100, 1030, 981, 803, and 737; ^1^H NMR (DMSO-*d*_6_, 400 MHz): *δ* 3.88 (s, 6H), 4.82 (s, 4H), 7.00 (d, 2H, *J* = 8.8 Hz), 7.23 (d, 2H, *J* = 16.1 Hz), 7.30 (dd, 2H, *J*_1_ = 1.5 Hz, *J*_2_ = 6.6 Hz), 7.45 (d, 2H, *J* = 15.4 Hz), 7.61 (d, 2H, *J* = 7.3 Hz), 7.65-7.66 (m, 2H), 7.71 (d, 1H, *J* = 5.9 Hz), 7.75 (d, 1H, *J* = 8.1 Hz), 7.95 (d, 2H, *J* = 8.8 Hz), 8.64 (brs, 2H), and 10.67 (s, 2H); TOF-MS* m/z*: 683.1911 (M + 1), calculated for C_35_H_30_N_4_O_11_: 682.1877.

#### 3.4.10. 2, 2′-((((1E,4E)-3-Oxopenta-1,4-diene-1,5-diyl)bis(2-methoxy-4,1-phenylene))bis(oxy))bis(N-(2,6-dimethylphenyl)acetamide) **3j**

Yield 87% (yellow solid); m.p. 216-217°C; IR (KBr film) *υ*_max⁡_ cm^−1^: 3390, 3246, 3019, 2921, 1669, 1618, 1589, 1510, 1469, 1338, 1253, 1142, 1098, 1033, 982, 852, and 771; ^1^H NMR (DMSO-*d*_6_, 400 MHz): *δ* 2.13 (s, 12H), 3.87 (s, 6H), 4.80 (s, 4H), 7.04 (d, 8H, *J* = 7.3 Hz), 7.25 (d, 2H, *J* = 16.1 Hz), 7.33 (dd, 2H, *J*_1_ = 2.2 Hz, *J*_2_ = 6.6 Hz), 7.45 (d, 2H, *J* = 2.2 Hz), 7.70 (d, 2H, *J* = 16.1 Hz), and 9.42 (brs, 2H); TOF-MS* m/z*: 649.2836 (M + 1), calculated for C_39_H_40_N_2_O_7_: 648.2734.

#### 3.4.11. 2, 2′-((((1E,4E)-3-Oxopenta-1,4-diene-1,5-diyl)bis(2-methoxy-4,1-phenylene))bis(oxy))bis(N-(4-chlorophenyl)acetamide) **3k**

Yield 87% (yellow solid); m.p. 117–119°C; IR (KBr film) *υ*_max⁡_ cm^−1^: 3371, 2937, 1693, 1644, 1593, 1509, 1465, 1261, 1142, 1093, 1034, 978, 826, and 720; ^1^H NMR (DMSO-*d*_6_, 400 MHz): *δ* 3.87 (s, 6H), 4.67 (s, 4H), 6.98 (d, 2H, *J* = 8.8 Hz), 7.23 (d, 2H, *J* = 15.4 Hz), 7.37 (d, 5H, *J* = 8.8 Hz), 7.44 (s, 3H), 7.64 (d, 4H, *J* = 8.8 Hz), and 7.68 (d, 2H, *J* = 15.4 Hz); TOF-MS* m/z*: 661.1430 (M + 1), calculated for C_35_H_30_Cl_2_N_2_O_7_: 660.1375.

#### 3.4.12. 2, 2′-((((1E,4E)-3-Oxopenta-1,4-diene-1,5-diyl)bis (4,1-phenylene))bis(oxy))bis(N-benzylacetamide) **3l**

Yield 87% (yellow solid); m.p. 278-279°C; IR (KBr film) *υ*_max⁡_ cm^−1^: 3371, 3281, 2919, 1671, 1655, 1585, 1509, 1423, 1317, 1232, 1175, 1058, 988, 831, and 750; ^1^H NMR (DMSO-*d*_6_, 400 MHz): *δ* 4.34 (d, 4H, *J* = 5.9 Hz), 4.62 (s, 2H), 7.04 (d, 4H, *J* = 8.8 Hz), 7.19 (d, 1H, *J* = 8.1 Hz), 7.22 (d, 4H, *J* = 8.1 Hz), 7.23 (d, 3H, *J* = 6.6 Hz), 7.28 (d, 3H, *J* = 7.3 Hz), 7.30 (d, 1H, *J* = 7.3 Hz), 7.72 (d, 2H, *J* = 15.4 Hz), 7.74 (d, 4H, *J* = 8.8 Hz), and 8.68 (t, 2H, *J* = 5.9 Hz); TOF-MS* m/z*: 561.2311 (M + 1), calculated for C_35_H_32_N_2_O_5_: 560.2456.

#### 3.4.13. 2, 2′-((((1E,4E)-3-Oxopenta-1,4-diene-1,5-diyl)bis(4,1-phenylene)) bis(oxy))bis(N-(4-methylbenzyl)acetamide) **3m**

Yield 87% (yellow solid); m.p. 218–220°C; IR (KBr film) *υ*_max⁡_ cm^−1^: 3326, 3047, 2915, 2839, 1658, 1593, 1538, 1511, 1423, 1335, 1293, 1250, 1175, 1062, 1031, 982, 832, and 753; ^1^H NMR (DMSO-*d*_6_, 400 MHz): *δ* 2.25 (s, 6H), 4.28 (d, 4H, *J* = 5.9 Hz), 4.60 (s, 4H), 7.03 (d, 4H, *J* = 8.8 Hz), 7.09 (d, 4H, *J* = 8.1 Hz), 7.12 (d, 4H, *J* = 8.8 Hz), 7.19 (d, 2H, *J* = 16.1 Hz), 7.72 (d, 2H, *J* = 16.1 Hz), 7.74 (d, 2H, *J* = 8.8 Hz), and 8.62 (t, 2H, *J* = 5.9 Hz); TOF-MS* m/z*: 589.2624 (M + 1), calculated for C_37_H_36_N_2_O_5_: 588.2658.

#### 3.4.14. 2, 2′-((((1E,4E)-3-Oxopenta-1,4-diene-1,5-diyl)bis(4,1-phenylene))bis(oxy))bis(N-(4-methoxybenzyl)acetamide) **3n**

Yield 87% (yellow solid); m.p. 255–257°C; IR (KBr film) *υ*_max⁡_ cm^−1^: 3326, 3037, 2915, 2839, 1658, 1598, 1538, 1511, 1423, 1335, 1293, 1250, 1176, 1113, 1062, 1031, 982, 832, and 753; ^1^H NMR (DMSO-*d*_6_, 400 MHz): *δ* 3.70 (s, 6H), 4.26 (d, 4H, *J* = 8.8 Hz), 4.59 (s, 4H), 6.85 (d, 4H, *J* = 8.8 Hz), 7.03 (d, 4H, *J* = 8.8 Hz), 7.16 (d, 4H, *J* = 8.1 Hz), 7.19 (d, 2H, *J* = 16.1 Hz), 7.71 (d, 2H, *J* = 16.1 Hz), 7.73 (d, 4H, *J* = 8.8 Hz), and 8.60 (t, 2H, *J* = 5.9 Hz); ^13^C NMR (DMSO-*d*_6_, 100 MHz): *δ* 41.29, 55.02, 66.97, 113.63, 115.24, 123.85, 127.99, 128.62, 130.21, 131.19, 142.10, 158.21, 159.57, 167.26, and 188.21; TOF-MS* m/z*: 621.2523 (M + 1), calculated for C_37_H_36_N_2_O_7_: 620.2453.

#### 3.4.15. 2, 2′-((((1E,4E)-3-Oxopenta-1,4-diene-1,5-diyl)bis(2-methoxy-4,1-phenylene))bis(oxy))bis(N-(4-methylbenzyl)acetamide) **3o**

Yield 87% (yellow solid); m.p. 209–211°C; IR (KBr film) *υ*_max⁡_ cm^−1^: 3332, 3268, 3074, 2922, 2837, 1662, 1592, 1547, 1510, 1465, 1310, 1256, 1196, 1166, 1138, 1032, 978, 852, 801, 762; ^1^H NMR (CDCl_3_, 400 MHz): *δ* 2.33 (s, 6H), 3.78 (s, 6H), 4.49 (d, 4H, *J* = 5.9 Hz), 4.62 (s, 4H), 6.92 (d, 2H, *J* = 8.1 Hz), 6.96 (d, 2H, *J* = 16.1 Hz), 7.10 (d, 2H, *J* = 2.2 Hz), 7.14 (d, 2H, *J* = 8.1 Hz), 7.15 (d, 5H, *J* = 3.7 Hz), 7.18 (d, 2H, *J* = 5.9 Hz), 7.21 (d, 3H, *J* = 2.2 Hz), 7.67 (d, 2H, *J* = 16.1 Hz); TOF-MS* m/z*: 649.2836 (M + 1), calculated for C_39_H_40_N_2_O_7_: 648.2759.

#### 3.4.16. 2, 2′-((((1E,4E)-3-oxopenta-1,4-diene-1,5-diyl)bis(2-methoxy-4,1-phenylene))bis(oxy))bis(N-(4-methoxybenzyl) acetamide) **3p**

Yield 87% (yellow solid); m.p. 167–169°C; IR (KBr film) *υ*_max⁡_ cm^−1^: 3417, 3286, 3051, 2931, 2835, 1655, 1586, 1512, 1464, 1305, 1248, 1185, 1145, 1031, 972, 841, 080, and 768; ^1^H NMR (DMSO-*d*_6_, 400 MHz): *δ* 3.71 (s, 6H), 3.84 (s, 6H), 4.26 (d, 4H, *J* = 5.9 Hz), 4.59 (s, 4H), 6.86 (d, 5H, *J* = 8.1 Hz), 6.96 (d, 2H, *J* = 8.1 Hz), 7.17 (d, 5H, *J* = 8.1 Hz), 7.24 (d, 2H, *J* = 16.1 Hz), 7.29 (dd, 2H, *J*_1_ = 1.5 Hz, *J*_2_ = 6.6 Hz), 7.69 (d, 2H, *J* = 16.1 Hz), and 8.40 (t, 2H, *J* = 5.9 Hz); ^13^C NMR (DMSO-*d*_6_, 100 MHz): *δ* 41.39, 55.05, 55.73, 68.02, 111.12, 113.66, 113.92, 122.79, 124, 128.59, 128.68, 131.07, 142.46, 149.52, 158.26, 167.38, and 188.11; TOF-MS* m/z*: 681.2734 (M + 1), calculated for C_39_H_40_N_2_O_9_: 680.2652.

### 3.5. *In Vitro* Cytotoxicity

KBM5 and HCT116 were used for anticancer assay. The cytotoxic effect of C-5 curcumin analogues was determined by MTT assay [34]. Briefly, HCT116 and KBM5 cells (5 × 10^4^ cells/mL) were treated with 5 *μ*M of indicated test sample in a final volume of 0.1 mL at 37°C for 72 h. Thereafter, 1 mg/mL of MTT solution was added to the untreated/treated cells. After 2 h incubation at 37°C, 0.1 mL of the cell lysis buffer (20% SDS; 50% dimethylformamide; pH 4.7) was added. After an overnight incubation at 37°C, the OD at 590 nm were measured using a 96-well multiscanner autoreader (Dynatech MR 5000, Chantilly, VA), with the extraction buffer as a blank and reduction in viability as per treatment was calculated by comparing with untreated cells as control.

### 3.6. Assessment of Anti-Inflammatory Potential: Electrophoretic Mobility Shift Assay

To determine the anti-inflammatory potential of curcumin analogues, downmodulation in NF-*κ*B activation was measured in untreated and treated KBM5 cells. Electrophoretic mobility shift assay (EMSA) was performed with nuclear extract of treated-, untreated-, and induced-cells as described previously [35]. In brief, nuclear extracts prepared from cancer cells were incubated with ^32^P end-labeled 45-mer double-stranded NF-*κ*B oligonucleotide (15 *μ*g of protein with 16 fmol of DNA) from the HIV long terminal repeat (5′-TTGTTACAAGGGACTTTC CGCTG GGGACTTTC CAGGGA GGCGT GG-3′, with NF-*κ*B-binding sites) for 30 min at 37°C. The resulting protein-DNA complex was separated from free oligonucleotides on 6.6% polyacrylamide gels. The dried gels were visualized by Phosphor-Imager imaging device (Molecular Dynamics, Sunnyvale, CA), and radioactive bands were quantified using Image Quant software.

## 4. Conclusion

These new curcumin analogues exhibited good potential to inhibit TNF-*α*-induced NF-*κ*B activation so they can be further optimised to get lead molecule with good anti-inflammatory activity. Some of these compounds also exhibited potent cytotoxicity against KBM5 and HCT116 cancer cell lines so further modifications of these molecules could be done to get a lead molecule for further studies.

## Figures and Tables

**Table 1:** Inhibition of hybrid molecules (**3a–3p**) on chronic myeloid leukemia (KBM5) and colon cancer (HCT116) cell lines at 5 *μ*M.

Compound	Percentage growth inhibition KBM5	Percentage growth inhibition HCT116	Activity
**3a**	76.06 ± 1.45	52.71 ± 1.01	High
**3b**	85.52 ± 0.66	57.88 ± 0.45	High
**3c**	29.38 ± 2.14	20.36 ± 1.48	Moderate
**3d**	36.32 ± 4.30	25.17 ± 2.98	Moderate
**3e**	30.09 ± 3.01	20.85 ± 2.08	Moderate
**3f**	16.33 ± 4.84	11.32 ± 3.36	Low
**3g**	8.65 ± 7.12	5.99 ± 4.94	Low
**3h**	86.76 ± 3.09	60.13 ± 2.14	High
**3i**	17.67 ± 1.54	12.25 ± 1.07	Low
**3j**	87.61 ± 3.43	60.72 ± 2.38	High
**3k**	75.09 ± 2.90	52.04 ± 2.01	High
**3l**	22.33 ± 3.82	15.48 ± 2.65	Moderate
**3m**	13.27 ± 2.08	9.20 ± 1.44	Low
**3n**	23.27 ± 1.84	16.12 ± 1.28	Moderate
**3o**	10.89 ± 4.25	7.55 ± 2.95	Low
**3p**	83.63 ± 0.34	57.96 ± 0.24	High
Control	0.00 ± 3.36	0.00 ± 2.33	
Curcumin	46.00 ± 1.49	46.87 ± 1.03	

Compounds are classified based on potential to inhibit growth of KBM5 cancer cell lines at 5 *μ*M, >60%; high activity, >20%; moderate activity, <20%; low activity.

**Table 2:** Molinspiration calculations of new curcumin analogues (**3a–3p**).

Compound	Molecular properties calculations						Drug likeness properties predictions					
	M.W.	*m*_*i*_log⁡*P*	TPSA Å^2^	OH–NH interaction	N violation	Vol.	GPCR	ICM	KI	NRL	PI	EI
**3a**	532	5.905	93	2	2	486	−0.12	−0.50	−0.25	−0.12	−0.08	−0.17
**3b**	601	7.213	93	2	2	513	−0.17	−0.65	−0.35	−0.24	−0.12	−0.29
**3c**	601	7.261	93	2	2	513	−0.16	−0.65	−0.35	−0.23	−0.11	−0.41
**3d**	690	7.523	93	2	2	522	−0.22	−0.69	−0.37	−0.29	−0.15	−0.30
**3e**	568	6.232	93	2	2	496	−0.16	−0.66	−0.33	−0.21	−0.10	−0.27
**3f**	588	5.634	93	2	2	552	−0.28	−0.88	−0.50	−0.39	−0.19	−0.17
**3g**	622	5.775	185	2	3	5.33	−0.45	−1.11	−0.75	−0.66	−0.26	−0.60
**3h**	592	5.085	112	2	2	537	−0.27	−0.88	−0.49	−0.42	−0.15	−0.42
**3i**	682	4.954	263	2	2	584	−0.85	−1.74	−1.28	−1.25	−0.56	−1.13
**3j**	648	4.814	112	2	1	603	−0.59	−1.42	−0.94	−0.89	−0.40	−0.82
**3k**	661	6.440	112	2	2	564	−0.39	−1.11	−0.69	−0.63	−0.25	−0.61
**3l**	560	5.307	93	2	2	520	−0.11	−0.61	−0.34	−0.29	0.02	−0.22
**3m**	588	6.204	93	2	2	553	−0.22	−0.84	−050	−0.46	−0.05	−0.39
**3n**	620	5.421	112	2	2	571	−0.34	−1.06	−0.68	−0.66	−0.12	−0.55
**3o**	648	5.384	112	2	2	604	−0.54	−1.39	−0.94	−0.95	−0.28	−0.81
**3p**	680	4.601	130	2	2	622	−0.74	−1.68	−1.21	−1.23	−0.41	−1.04
Curcumin	368	2.303	93	2	0	332	−0.06	−0.20	−0.26	0.12	−0.14	−0.08

GPCRL: GPCR ligand; ICM: ion channel modulator; KI: kinase inhibitor; NRL: nuclear receptor ligand; PI: protease inhibitor; EI: enzyme inhibitor.

**Table 3:** Osiris calculations of new curcumin analogues (**3a–3p**).

Compound	Prediction of toxicity risk				Molecular properties calculations				
	MUT	TUMO	IRRI	REP	M.W.	*C*log⁡*P*	log⁡*S*	D-L	D-S
**3a**	G	G	G	G	532	5.04	−6.57	2.13	0.29
**3b**	G	G	G	G	600	6.26	−8.04	3.80	0.19
**3c**	G	G	G	G	600	6.26	−8.04	4.59	0.20
**3d**	G	G	G	G	688	6.43	−8.24	1.80	0.16
**3e**	G	G	G	G	568	5.15	−7.20	2.53	0.25
**3f**	G	G	G	G	588	6.30	−7.95	5.78	0.20
**3g**	Y	Y	G	G	622	4.78	−7.49	−1.47	0.09
**3h**	G	G	G	G	592	4.83	−6.61	3.27	0.28
**3i**	Y	Y	G	G	682	4.57	−7.53	−0.33	0.11
**3j**	G	G	G	G	648	6.09	−7.98	6.77	0.19
**3k**	G	G	G	G	660	6.05	−8.08	5.70	0.18
**3l**	G	G	G	G	560	4.61	−6.20	3.91	0.32
**3m**	G	G	G	G	588	5.24	−6.89	2.97	0.25
**3n**	G	G	G	G	620	4.40	−6.24	4.60	0.33
**3o**	G	G	G	G	480	5.03	−6.93	4.18	0.24
**3p**	G	G	G	G	680	4.19	−6.27	5.88	0.28
Curcumin	G	G	G	G	368	2.97	−3.62	−3.95	0.39

G = no toxicity risk; Y = low toxicity risk; R = high toxicity risk; MUT: mutagenic; TUMO: tumorigenic; IRRI: irritant; REP: reproductive effective; Mol. Wt.: molecular weight in g/mol; *C*log⁡*P*: log of octanol/water partition coefficient; *S*: solubility; D-L: drug likeness; D-S: drug score.
